# Responses of non-structural carbohydrate and carbon, nitrogen, and phosphorus chemometrics in needles of early shaded *Pinus yunnanensis* seedlings to drought

**DOI:** 10.1186/s12870-025-06265-8

**Published:** 2025-03-01

**Authors:** Chengyao Liu, Junwen Wu, Jianyao Gu, Huaijiao Duan

**Affiliations:** 1 College of Forestry, Southwest Forestry University, Kunming, 650224 Yunnan China; 2 The Key Laboratory of Forest Resources Conservation and Utilization in the Southwest Mountains of China, Ministry of Education, Southwest Forestry University, Kunming, 650224 China; 3 Key Laboratory of National Forestry and Grassland Administration on Bioaffiliationersity Conservation in Southwest China, Southwest Forestry University, Kunming, 650224 China

**Keywords:** *Pinus yunnanensis*, Light intensity, Drought, Non-structural carbohydrates, C, N, P stoichiometry

## Abstract

With global warming, the frequency and duration of drought is becoming longer and longer, which seriously affects the survival of trees. Light intensity control, such as shading, is an important measure in seedling nurseries. However, it is unclear whether early shading affects the drought tolerance of seedlings used in afforestation. We conducted a two-stage experiment on *Pinus yunnanensis* seedlings. First, three different shading treatments were set, namely HL (0% shading), ML (55% shading), and LL (80% shading). After 90 days of cultivation, the seedlings of each shading treatment were subjected to CK (water content of 90% ± 5%), LD (water content of 75% ± 5%), MD (water content of 60% ± 5%) and SD (water content of 45% ± 5%) continuous drought for 30 days. The contents of non-structural carbohydrates (NSCs), carbon (C), nitrogen (N), and phosphorus (P) and their ratios in the needles of *P. yunnanensis* seedlings were measured. Early shading affected the starch accumulation and the balance between C absorption and consumption in *P. yunnanensis* seedlings during drought. Early shading affected C consumption, P utilization efficiency, and N restriction under drought stress. The phenotypic plasticity index showed that the plasticity of *P. yunnanensis* seedlings under drought stress followed the order: LL > HL > ML. The results of principal component analysis showed that the performance under drought stress followed the order HL > LL > ML. These results indicated that early shading could affect the response of *P. yunnanensis* seedlings to drought. The *P. yunnanensis* seedlings grown under HL and LL were more resistant to drought stress than those grown under ML. It is suggested that 0% or 80% shading should be applied at seedling stage to improve the drought resistance of *P. yunnanensis*.

## Introduction

Nowadays, the frequency and duration of drought in the world are becoming longer and longer due to global warming, which seriously affects the survival of trees [1]. The most direct effect of drought on plant survival and growth is water loss [2]. Light is an important environmental factor that affects the growth and distribution of plants and determines their photosynthesis and vitality [3]. An optimal light intensity can help maintain plant morphological structure, photosynthesis, and physiological metabolism, and therefore can promote the growth and development of forest plants and forest regeneration, ensuring the ecological and economic benefits of plants [4, 5]. In plantations, management practices, such as tending, thinning, and selective cutting, are commonly used to adjust the spatial structure of the stand and improve the light intensity and other conditions to promote tree growth [6]. However, in it unclear how to best alter the light conditions to promote stress resistance. If seedlings are subjected to drought stress, the cell wall damage cannot be repaired, which will lead to seedling death and therefore reduce the effectiveness of afforestation. Therefore, before afforestation, seedlings need to be treated to improve their quality and stress resistance [7]. Subjecting seedlings to shading and drought stress treatments can improve their growth after afforestation. However, the effect of shading treatments on the drought resistance of seedlings after transplanting is not clear.

Non-structural carbohydrates (NSCs), including soluble sugars and starches, are key indicators of plant physiological activity and responses to environmental changes; NSCs play an important role in maintaining normal plant growth and plant resistance to environmental stress [8]. Both drought and shading can lead to an imbalance in the carbon supply and demand in plants, affect the balance between plant growth and NSC storage, and thus affect plant stress resistance [9, 10]. Under drought stress, starch in plants can be converted into soluble sugar to resist adversity [11]. Under shading stress, the NSC content of *Quercus aliena* seedlings was significantly lower than that under natural light [12]. Carbon (C), nitrogen (N), and phosphorus (P) are essential elements for plant growth and physiological activities. Plants adapt to environmental changes by altering the contents of these elements; therefore, these contents can reflect the relationship between plants and the environment [13, 14]. Under drought stress, plant contents and ratios of C, N, and P are altered in different ways [15, 16]. Drought can affect the fixation of C and the absorption of N and P, resulting in a reduction in C, N, and P contents in plants [17, 18]. Shading can also significantly affect physiological and ecological processes, such as the accumulation, absorption, and distribution of C, N, and P in plants [19]. For example, the proportion of N and P in *Medicago sativa* L. increased under a suitable light intensity [20]. To date, research on the changes and distribution patterns of NSC, C, N, and P in trees has mainly focused on drought and shading. However, with the implementation of thinning and selective cutting, the light intensity in the stand has changed, and the concentration changes and distribution patterns of NSC, C, N and P in trees in response to drought have rarely been reported.

Leaves are the main photosynthetic organs of plants; however, they are very sensitive to environmental changes [21]. The contents of C, N, and P in leaves are related to many key plant growth and ecosystem functions and therefore can be used as indicators to evaluate plant nutrient utilization and resistance to environmental changes [22]. NSCs are fixed by photosynthesis in plant leaves and are important for plant growth, development, and reproduction. NSCs in plant leaves also have important functions in metabolism, water transportation, assimilate and osmotic adjustment, and resistance to external biological and environmental stresses [23]. Therefore, examining the C, N, P, and NSC contents in leaves can reveal the responses and adaptations of tree growth and eco-physiological processes to multi-factor environmental changes [24].

*Pinus yunnanensis* is a native tree species in southwest China, which has high economic and ecological value [25]. In recent years, global warming has increased temperatures and caused frequent drought events in southwest China, resulting in the death of many *P. yunnanensis* trees [2]. *P. yunnanensis* is susceptible to drought stress, especially at the seedling stage, and is very sensitive to water changes in the surface soil, which seriously affects its growth and development [26]. But in the management of *P. yunnanensis* plantation, density control, pruning and thinning can affect the change of light intensity [27]. This raises a question: do different light environments in the early growth stage affect the ability of *P. yunnanensis* to adapt to drought? That is, can early shading reduce the negative effects of drought? In this study, we analyzed the drought responses of NSC, C, N, and P contents and their ratios in the needles of *P. yunnanensis* seedlings cultured under different light intensities. Our results provide data and a theoretical basis for the management of light during cultivation of *P. yunnanensis* seedlings in containers.

## Materials and methods

### Study site and test materials

The experiment was conducted in the Southwest Forestry University Arboretum (Kunming, Yunnan, 25°03′ N, 102°46′ E), located in the monsoon region of the subtropical plateau, with an altitude of 1964 m and a mild climate. The annual frost-free period was about 240 days, the annual mean temperature was 16.5 °C, the annual average precipitation was 1035 mm, and the annual average relative humidity was 67%. The soil type in this area is red loam. The temperature in the greenhouse was 18–37 °C, and the relative humidity of the air was 22–47%.

### Experimental material

One-year-old *P. yunnanensis* seedlings were used as the experimental materials. On August 1, 2021, an improved variety of *P. yunnanensis* (improved variety number: Yun R-SS-PY-035-2020) was selected by the early project team in Malonghe Forest Farm, Shuangbai County Province. After eight months of cultivation at Malonghe Forest Farm in Shuangbai County, the improved variety was transferred to the Southwest Forestry University Arboretum in April 2022 for transplanting and cultivation. The seedlings of *P. yunnanensis* were transplanted into plastic pots with a diameter of 20.5 cm, ground diameter of 14.5 cm, and height of 18.5 cm. One *P. yunnanensis* seedling was planted in each pot, which was packed with 3.5 kg of soil mixed with pine needle nutrient humus (Songming County of Kunming city) at a ratio of 3:2. A tray was placed underneath each pot and pots were placed on the ground of the greenhouse to ensure good ventilation. After transplanting, the soil moisture content was immediately adjusted to the field water holding capacity to ensure the survival of the seedlings. The *P. yunnanensis* seedlings were grown for 3 months under the different treatments (see below). In July, 450 seedlings were selected for analysis. Shading and soil water were controlled artificially to ensure consistent experimental treatments. During the experiment, the position of potted plants was randomly changed periodically to reduce the influence of microclimate on seedlings.

### Experimental design

Shading treatment: Three shading treatments were established using black shading nets with different densities. The treatments were 55% (ML) and 80% (LL) shade (Simulation of understory and gap environment with different light transmittance in field) and unshaded full light (HL) shading (Simulating bare ground in the wild) lasted for 90 days, from June 28 to September 28.

Drought treatment: After shading, *P. yunnanensis* seedlings under each shading treatment were divided into four groups corresponding to four drought treatments: Reference to other local scholars drought stress classification method [28]. normal moisture (CK), light drought (LD), medium drought (MD), and severe drought (SD). The treatments were established by adjusting the moisture content in the pots to 90% ± 5%, 75% ± 5%, 60% ± 5%, and 45% ± 5% of field water holding capacity, respectively, which corresponded to an actual water content of 21.38–19.13%, 18–15.75%, 14.63–12.38%, and 11.25–9%, respectively. Drought treatments lasted 30 days, from October 1 to October 30, 2022. The soil moisture was measured using a soil moisture meter and controlled using the weighing method. The potted plants were weighed at 17:00 each day and were controlled or watered according to the target weight. Plants were sampled after the drought treatment.

### Measurement of indicators

Five healthy needles from *P. yunnanensis* seedlings were weighed in each treatment and put into an oven at 120 °C for 30 min. Then, the needles were dried at 80 °C for 48 h until constant weight (Weigh twice in a row and its mass will not change) and then ground. The contents of soluble sugar and starch were determined using the anthrone ethyl acetate-concentrated sulfuric acid method [29]. The total carbon content was determined by potassium dichromate sulphuric acid oxidation, the total nitrogen content was determined by Nessler’s colorimetry, and the total phosphorus content was determined by molybdenum antimony anticolorimetry [30].

### Data processing and analysis

Data pre-processing and visualization were completed in Excel 2016 and GraphPad Prism 9.5, respectively. All graphs present the mean ± standard deviation. SPSS 27.0 statistical analysis software was used to carry out one-way ANOVA analysis of variance to determine the effects of drought treatment under each shading treatment. Two-way ANOVA was used to analyze the effects of shading and drought on NSC, NSC components, C, N, and P contents in *P. yunnanensis* seedlings. Principal component analysis and mapping were performed in Origin 2021. The phenotypic plasticity index (P) was determined as:

P = (Xmax − Xmin) / Xmax.

where Xmax and Xmin represent the maximum and minimum values of each index, respectively.

## Results

### Effects of shading and drought on NSC and its components

Shading, drought, and their interaction significantly affected the contents of soluble sugar, starch, and total NSC, and the ratio of soluble sugar to starch in *P. yunnanensis* seedlings (*p* < 0.01; Table 1).

**Table 1:** Results from two-way ANOVA of shading, drought, and their interaction on NSC and its components in *P. yunnanensis* seedlings

Factor	Soluble sugar	Starch	NSC	Soluble sugar/Starch
Shade	198.473**	344.915**	516.728**	211.226**
Drought	2314.228**	463.248**	436.859**	1276.396**
Shade × Drought	837.221**	932.841**	895.405**	560.377**

* *p* < 0.05; ** *p* < 0.01.

**Fig. 1:**
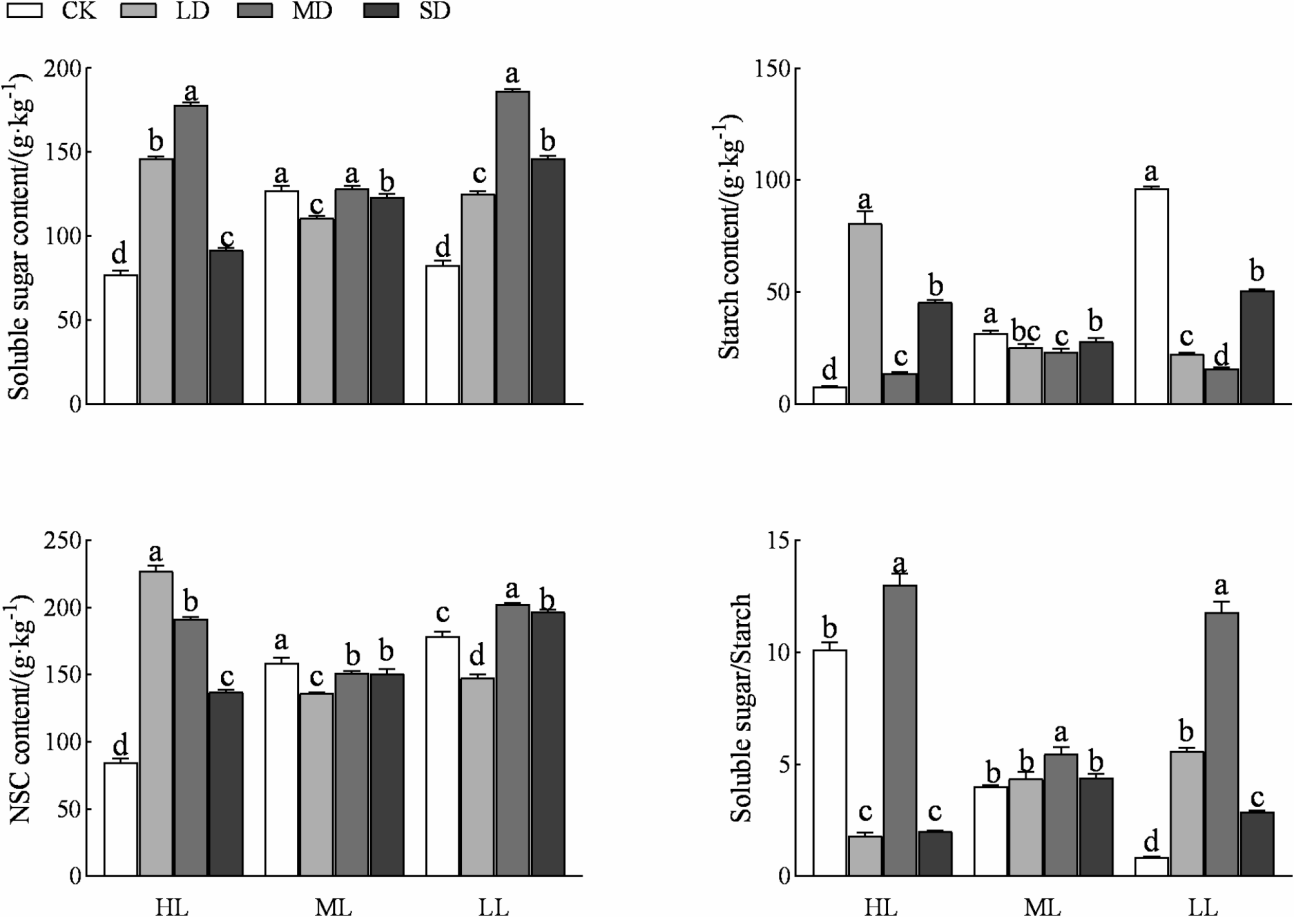
Drought responses of NSC and its components in *P. yunnanensis* seedlings cultured under different shading treatments. Different lowercase letters indicate significant differences between drought treatments within each shading treatment (*p* < 0.05). HL (shading 0%), ML (shading 55%), LL (shading 80%). CK (water content was 90% ± 5%), LS (water content was 75% ± 5%), MS (water content was 60% ± 5%), and SS (water content was 45% ± 5%)

The contents of NSC and its components in *P. yunnanensis* seedlings cultured under different light intensities showed different trends under different drought treatments (*p* < 0.05; Fig. 1). Compared with HL and LL treatments, the ML treatment resulted in smaller changes with increasing drought intensity. The soluble sugar content of seedlings under each shading treatment reached the maximum under the MD treatment. The soluble sugar content of seedlings treated with HL and LL was the lowest under the CK treatment, whereas that of seedlings treated with ML was the lowest under the LD treatment. The starch content of HL seedlings was the highest in the LD treatment and the lowest in the CK treatment; however, the starch content of ML and LL seedlings reached the highest in the CK treatment and the lowest in the MD treatment. The NSC content of HL seedlings was the highest under the LD treatment and the lowest under the CK treatment. The NSC content of ML seedlings was the highest in the CK treatment and the NSC content of LL seedlings was the highest in the MD treatment, but the NSC contents of both ML and LL seedlings were the lowest under the LD treatment. The soluble sugar/starch ratios of HL, ML, and LL seedlings were the highest under the MD treatment, the ratio of the HL seedlings was the lowest under the LD treatment, and ratios of the ML and LL seedlings were the lowest under CK treatment.

### Effects of shading and drought on C, N, P, and their ratios

Shading, drought, and their interaction had highly significant effects on the C, P, and C/N ratio of *P. yunnanensis* seedlings (*p* < 0.01; Table 2). Shading and the interaction of shading and drought had highly significant effects on N and C/P, and drought had a significant effect on N and C/P (*p* < 0.05). Shading and the interaction of shading and drought had a significant effect on N/P, whereas drought had no effect on N/P.

**Table 2:** Results of two-way ANOVA of shading, drought, and their interaction on the C, N, and P contents and their ratios in *P. yunnanensis* seedlings

Factor	C	*N*	*P*	C/*N*	C/*P*	*N*/*P*
Shade	16.619**	13.321**	262.028**	17.055**	13.576**	6.589*
Drought	18.286**	3.858*	314.892**	11.224**	4.208*	1.784
Shade × Drought	22.447**	7.279**	1063.935**	5.556**	91.137**	45.271**

* *p* < 0.05; ** *p* < 0.01.

**Fig. 2:**
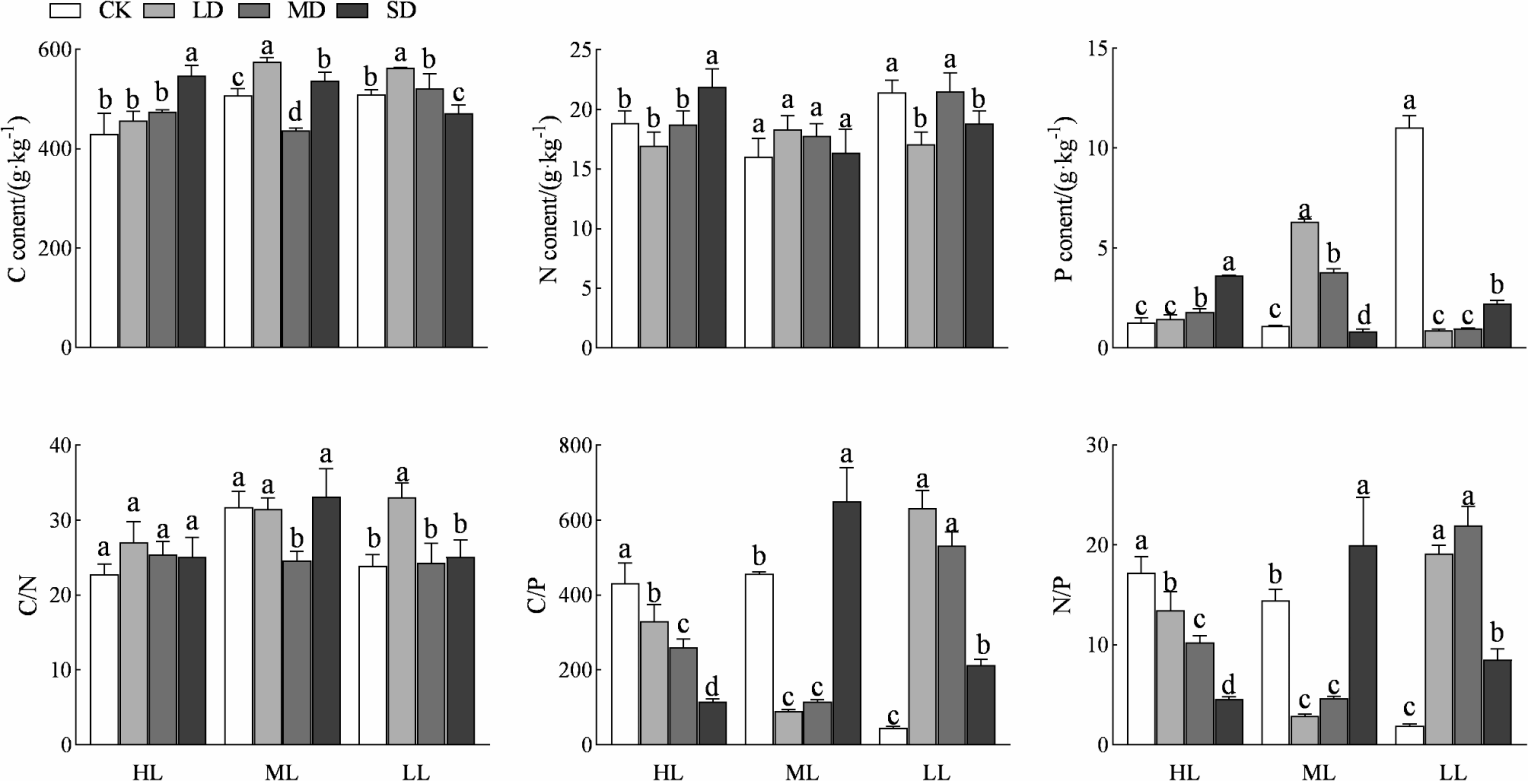
Drought responses of C, N, P and their ratios in *P. yunnanensis* seedlings cultured under different shading treatments. Different lowercase letters indicate significant differences between drought treatments within each shading treatment (*p* < 0.05). HL (shading 0%), ML (shading 55%), LL (shading 80%). CK (water content was 90% ± 5%), LS (water content was 75% ± 5%), MS (water content was 60% ± 5%), and SS (water content was 45% ± 5%)

The C, N, and P contents and their ratios in *P. yunnanensis* seedlings cultured under different light intensities showed different trends under different drought stress (*p* < 0.05; Fig. 2). The C content of HL seedlings increased with increasing drought degree, while the C content of ML and LL seedlings reached the maximum under LD, when it was significantly higher than that under CK. The N content in HL seedlings first decreased and then increased with increasing drought degree, reaching the maximum under the SD treatment when it was significantly higher than that under CK. The N content in ML seedlings did not differ significantly among different drought treatments. The N content of LL seedlings reached the maximum under the MD treatment. The P content of HL seedlings increased with increasing drought degree, while that of ML seedlings first increased and then decreased, reaching the maximum under LD when it was significantly higher than that under CK. The content of P in LL seedlings first decreased and then increased with increasing drought degree.

The C/N ratio of HL seedlings did not differ significantly among different drought treatments. The C/N ratio of ML seedlings was significantly lower under MD than under CK. The C/N ratio of LL seedlings reached the maximum under LD when it was significantly higher than that under CK (*p* < 0.05). The C/P and N/P ratios of HL seedlings decreased with increasing drought degree. The C/P and N/P ratios of ML seedlings first decreased and then increased with increasing drought degree and were significantly higher under SD than under CK. The C/P and N/P ratios of LL seedlings were significantly higher under LD, MD, and SD than under CK.

### Phenotypic plasticity index analysis

**Fig. 3:**
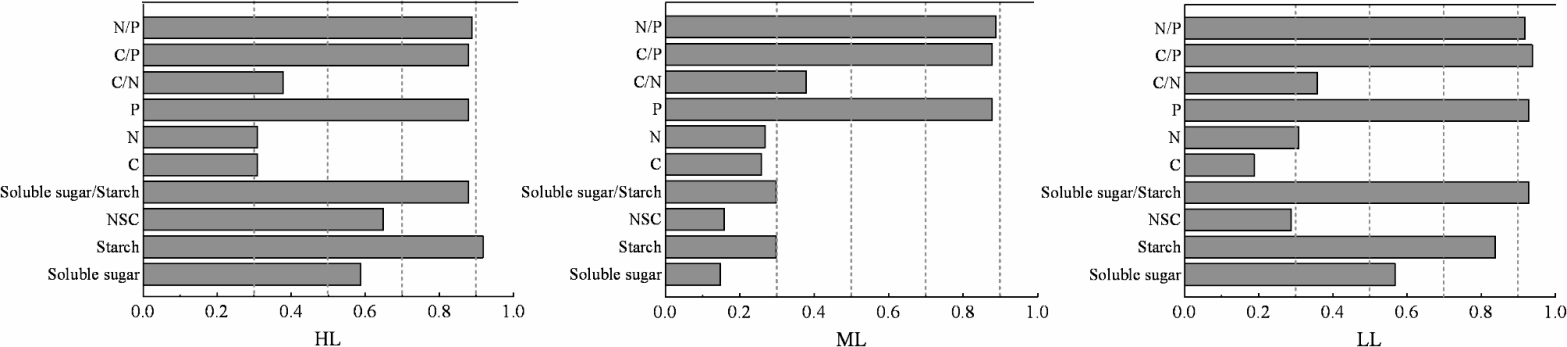
Phenotypic plasticity index in response to drought for needle NSC, C, N, and P and their ratios in *P. yunnanensis* seedlings cultivated under different shading treatments. HL (shading 0%), ML (shading 55%), LL (shading 80%)

Plasticity indices for each variable under each shading treatment are shown in Fig. 3. In HL seedlings, the plasticity indices for starch (0.92), N/P (0.89), P (0.88), soluble sugar starch (0.88), and C/P (0.88) were high while those for C (0.31) and N (0.31) were low. In ML seedlings, the plasticity indices for N/P (0.89), P (0.88) and C/P (0.88) were high while those for soluble sugar (0.15) and NSC (0.16) were low. In LL seedlings, the plasticity indices for C/P (0.94), P (0.93), soluble sugar/starch (0.93) and N/P (0.92) were high while that for C (0.19) was low.

### Principal component analysis

**Fig. 4:**
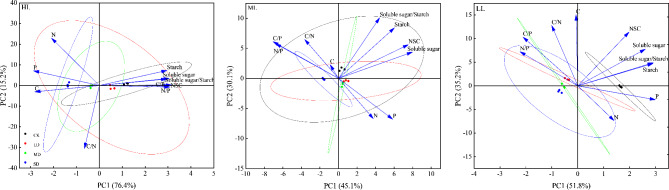
Principal component analysis of NSC and C, N, and P and their ratios in needles of *P. yunnanensis* seedlings under drought stress. Dot colors represent different drought stress intensities; ellipses denote the confidence interval of each parameter under different drought stress intensities; arrows represent the relationship between each index and the principal components. HL (shading 0%), ML (shading 55%), LL (shading 80%)

The NSC and C, N, and P stoichiometric characteristics of *P. yunnanensis* seedling needles after drought were analyzed by principal component analysis (Fig. 4). The cumulative contribution rates of the first two principal components for HL, ML, and LL seedlings were 91.6%, 75.2%, and 87%, respectively. For HL seedlings, NSC, N/P, soluble sugar, C/P, starch, and soluble sugar/starch had large weighted coefficients in PC1 and N had a large weighted coefficient in PC2. For ML seedlings, soluble sugar, NSC, starch, and P had large weighted coefficients in PC1 and soluble sugar/starch had a large weighted coefficient in PC2. For LL seedlings, P, soluble sugar/starch, starch, and soluble sugar had large weighted coefficients in PC1, and C and C/N had large weighted coefficients in PC2. Based on the cumulative contributions, HL seedlings performed better than LL seedlings, and LL seedlings performed better than ML seedlings.

## Discussion

### Effects of shading and drought on NSC and its components

NSC contents in plants are affected by changes in the external environment. When a plant is under stress, its NSC begins to hydrolyze as a buffer to adjust the plant’s state [31]. In this study, shading, drought, and their interactions had significant effects on NSC content and its components in *P. yunnanensis* seedlings (Table 1). Drought inhibits leaf photosynthesis, and leaves require a lot of soluble sugar to maintain normal cell tension [32]. In this study, the soluble sugar content of HL and LL seedlings followed a similar trend; the maximum soluble sugar content occurred under the MD treatment and was lower in the SD treatment. The effect of drought on the soluble sugar content in ML seedlings was smaller than that on HL and LL seedlings (Fig. 1). Studies have shown that starch content increases in arid environments [33]. In this study, the starch content in HL seedlings was significantly higher under drought stress than in the control, while the starch content in ML and LL seedlings was significantly lower under drought than in the control (Fig. 1). These results indicate that the shading in the early stage affected the accumulation of starch in the needles of *P. yunnanensis* seedlings during drought. NSC is the main intermediate product between photosynthesis (carbon absorption) and growth utilization (carbon consumption), and its storage size reflects the balance between carbon absorption and consumption [34]. In this study, the NSC content of *P. yunnanensis* seedlings under HL increased significantly under drought stress, while the NSC content of seedlings under ML decreased significantly under drought stress (Fig. 1). These results indicate that shading affected the balance between carbon uptake and consumption of *P. yunnanensis* seedlings in response to drought. Similarly, in their study of Heptacodium miconioides seedlings, Wei et al. found that the NSC content in leaves changed dynamically under shading [35]. Under drought stress, the change of soluble sugar/starch ratio can reflect the stress resistance of plants. Under drought stress, a part of starch in plants will be hydrolyzed into soluble sugar, resulting in the increase of soluble sugar/starch ratio [36]. In this study, the soluble sugar/starch ratio of *P. yunnanensis* seedlings treated with HL was significantly lower under LD and SD than under CK; the soluble sugar/starch ratio of ML seedlings first increased and then decreased with increasing drought degree but was lowest under CK; and the soluble sugar/starch ratio in LL seedlings was significantly higher under drought stress than under CK (Fig. 1). These results indicate that early shading could, to some extent, alleviate the drought stress.

### Effects of shading and drought on C, N, P, and their ratios

Plant contents of C, N and P can reflect plant adaptability or sensitivity to environmental change [37]. In this study, shade, drought and their interactions affected C, N, and P contents and their stoichiometry to varying degrees (Table 2). The C assimilated by photosynthesis is the substrate and energy source of various physiological and biochemical processes in plants [38]. In this study, the C content in needles of *P. yunnanensis* seedlings treated with HL increased with increasing drought (Fig. 2). Drought can affect the growth and nutrient absorption of seedlings and reduce the consumption of C. Wang et al. showed similar results for Caragana microphylla seedlings under drought conditions [39]. The needle C content of ML seedlings first increased, then decreased, and then increased with increasing drought degree, and the needle C content of LL seedlings first increased and then decreased with increasing drought degree. These results indicate that the early shading affected the C consumption of *P. yunnanensis* seedlings under drought. N and P are important components of plant photosynthesis enzymes. Changes in plant N and P can affect the photosynthesis rate and therefore plant growth [40]. In this study, the needle contents of N and P in HL seedlings were significantly higher under SD than under CK (Fig. 2). This indicates that severe drought stress affected nutrient transport, and N and P formed and accumulated in leaves. In LL seedlings, the needle N content first decreased, then increased, and then decreased with increasing drought degree, and the P content first decreased and then increased. This indicates that severe shading affected the photosynthesis and transpiration of needles, which altered the N and P contents of *P. yunnanensis* seedlings under drought.

The ratios of C to nutrient contents (i.e., C/N and C/P) reflect the ability of plants to assimilate C when absorbing N and P [41]. In this study, the C/N ratio of needles of *P. yunnanensis* seedlings under HL was not affected by drought treatment (Fig. 2), while the C/N ratio of ML seedlings was significantly lower under MD than under CK, and the C/N ratio of LL seedlings was significantly higher under LD than under CK. These results indicate that early shading affects the N utilization efficiency of seedlings in response to drought, thus affecting the C assimilation ability. The C/P ratio of HL seedlings decreased with increasing drought degree; the C/P ratio of ML seedlings was significantly higher under SD than under CK; and the C/P ratio of LL seedlings was significantly higher under drought treatments than under CK (Fig. 2). These results indicate that the degree of shading in the early stage could affect the P utilization efficiency of *P. yunnanensis* seedlings in response to drought. The N/P ratio can reflect the biological characteristics of plants, and it is also a key index to determine which element limits plant growth [42]. In this study, the N/P ratio of HL seedlings decreased with increasing drought degree. This may be because a lower soil moisture reduces the mineralization and availability of N in soil. Zhang et al. showed similar results for white Fraxinus malacophylla seedlings under drought conditions [43].

### Drought adaptation strategies of *P. yunnanensis* seedlings cultured under different shading treatments

Plants constantly adapt to their surrounding environment, eventually forming physiological and morphological adaptations [44]. Plant phenotypic plasticity is an important mechanism by which plants adapt to their environment and cope with adversity. Plasticity can reflect not only the growth and development of plants but also the ability of plants to adapt to their environment. The adaptability of plants to their environment is positively correlated with their plasticity index [45]. In this study, LL seedlings showed greater plasticity in response to drought stress than HL seedlings, while HL seedlings showed greater plasticity than ML seedlings (Fig. 3). This indicates that the seedlings of *P. yunnanensis* treated with LL shading have the greatest plasticity in response to drought stress. Principal component analysis showed that HL seedlings had a larger response to drought than LL seedlings, while LL seedlings had a larger response to drought than ML seedlings (Fig. 4). These results indicate that *P. yunnanensis* seedlings cultivated under HL and LL light are more resistant to drought stress than seedlings cultivated under ML light. In summary, 0% or 80% shading can be given to seedlings in the nursery cultivation of *P. yunnanensis* in arid areas to improve the drought resistance of *P. yunnanensis* in the later stage.

## Conclusions

This study explored the effects of shading during seedling cultivation on the drought resistance of *P. yunnanensis*. The contents of NSC and its components in *P. yunnanensis* seedlings cultivated under different light intensities showed different trends under different drought treatments. Shading during the early growth stage affects the accumulation of starch and the balance between carbon absorption and consumption during drought. Early shading affected C consumption of *P. yunnanensis* seedlings during drought. Early shading also affected the P utilization efficiency under drought stress and relieved the N limitation in seedlings under drought stress. The drought resistance of seedlings of *P. yunnanensis* cultivated under HL and LL light intensity was stronger than that under ML light intensity. In order to ensure the best growth effect of *P. yunnanensis*, it is suggested that 0% or 80% shading treatment should be carried out at seedling stage to improve drought resistance.

## Data Availability

No datasets were generated or analysed during the current study.

## References

[1] Chaves MM, Maroco JP, Pereira JS. Understanding plant responses to drought, from genes to the whole plant. Funct. Plant Biol. 2003;30:239–64. 10.1071/FP02076 32689007

[2] Liu CY, Wu JW, Gu JY, Duan HJ. Response of non-structural Carbohydrates and Carbon, Nitrogen, and Phosphorus Stoichiometry in *Pinus yunnanensis* seedlings to Drought Re-watering. Forests. 2024;15:1864.

[3] Massimo B, Krishnasamy M, Michael R, Roberto Z, Riccardo V, Namachevayam N. Low-night temperature increased the photoinhibition of photosynthesis in grapevine (*Vitis vinifera* L. Cv. Riesling) leaves. Environ exp bot. 2005;57:25–31.

[4] Chen B, Li HY, Liu YW, Xia B, Sun SW, Sun Y, He M. Effects of different light intensities on morphogenesis and ultrastructure of Gibasis pellucida leaf. J grs ind. 2019;28:175–85.

[5] Geoffrey GP, David RF, Irene CGS. Consequences of environmental heterogeneity for the photosynthetic light environment of a tropical forest. Agric Meteorol. 2019;278:107761.

[6] Russell SE, Liu H, Thistle H, Thistle H, Strom B, Greer M, Lamb B. Effects of thinning a forest stand on sub-canopy turbulence. Agric Meteorol. 2018;248:295–305.

[7] Luoranen J, Rikala R. Post-planting effects of early-season short-day treatment and summer planting on Norway spruce seedlings. Silva Fennica. 2015;49:1.

[8] Sun T, Mao ZJ, Dong LL. Further evidence for slow decomposition of very fine roots using two method: Litterbags and intact cores. Plant Soil. 2013;366:633–46.

[9] Liu YX, Jing HQ, Wu JW. Non-structural carbohydrate (NSC) content and C:N:P stoichiometry of *Pinus yunnanensis* seedling needles in response to shade treatment. Ind Crops Prod. 2024;210:118138.

[10] Zhao ZJ, Wang LN, Liu YX. Effects of drought on non-structural carbohydrates and C, N, and P stoichiometric characteristics of *Pinus yunnanensis* seedlings. J Res. 2024;35:94–106.

[11] Kannenberg SA, Phillips RP. Non-structural carbohydrate pools not linked to hydraulic strategies or carbon supply in tree saplings during severe drought and subsequent recovery. Tree Phytol. 2019;40:259–71. 10.1093/treephys/tpz132 31860721

[12] Chen ZC, Liu XJ, Liu C, Wan XZ. Responses of growth, photosynthesis and nonstructural carbohydrate of Q*uercus aliena var.acuteserrata* seedlings to shading and simulated sunfleck. J Ecol. 2017;36:935–43.

[13] Moe SJ, Stelzer RS, Forman MR. Recent advances in ecological stoichiometry: insights for population and community ecology. Oikos. 2005;109:29–39.

[14] Tian D, Reich BP, Chen YH. Global changes alter plant multi-element stoichiometric coupling. New Phytol. 2019;221:807–17. 30256426 10.1111/nph.15428

[15] He MZ, Dijkstra F. A. Drought effect on plant nitrogen and phosphorus: a meta-analysis. New Phytol. 2014;204:924–31. 25130263 10.1111/nph.12952

[16] Sainju UM, Lenssen AW, Ghimire RP. Root biomass, root /shoot ratio, and soil water content under perennial grasses with different nitrogen rates. Field Crop Res. 2017;210:183–91.

[17] Hassan MU, Aamer M, Chattha MU. The role of potassium in plants under drought stress: Mini review. J Basic Appl Sci. 2022;13:268–71.

[18] Sanaullah M, Rumpel C, Charrier X. How does drought stress influence the decomposition of plant litter with contrasting quality in a grassland ecosystem? Plant Soil. 2012;352:277–88.

[19] Dickman EM, Newell JM, González MJ, Vanni MJ. Light, nutrients, and food-chain length constrain planktonic energy transfer efficiency across multiple trophic levels. PNAS. 2008;105:18408–12. 19011082 10.1073/pnas.0805566105 PMC2587603

[20] Ma ZL, Yang WQ, Wu FZ, Gao S. Effects of shading on the aboveground biomass and stiochiometry characteristics of *Medicago sativa*. Chin J Appl Ecol. 2014;25:3319–44. 25898609

[21] Liu Z, Zhu Y, Li F, Jian GE. Non-destructively predicting leaf area, leaf mass and specific leaf area based on a linear mixed-effect model for broadleaf species. Ecol Indic. 2017;78:340–50.

[22] Qin J, Shangguan ZP, Xi WM. Seasonal variations of leaf traits and drought adaptation strategies of four common woody species in South Texas, USA. J Res. 2019;30:1715–25.

[23] Hartmann H, Ziegler W, Trumbore S. Lethal drought leads to reduction in nonstructural carbohydrates in Norway spruce tree roots but not in the canopy. Funct Ecol. 2013;27:413–27.

[24] Zheng YP, Wang HX, Lou X, Yang QP, Xu M. Advances in studies on non-structural carbohydrate changes and their influencing factors in woody plants. J Appl Ecol. 2014;25:1188–96. 25011317

[25] Zhou C, Gu X, Li J, Su X, Chen S, Tang JR, Chen L, Cai NH, Xu YL. Physiological characteristics and transcriptomic responses of *Pinus yunnanensis* lateral branching to different shading environments. Plants. 2024;13:1588. 38931020 10.3390/plants13121588 PMC11207258

[26] Marod D, Kutintara U, Tanaka H. Effects of drought and fire on seedling survival and growth under contrasting light conditions in a seasonal tropical forest. J Veg Sci. 2004;15:691–700.

[27] Wang LN, Wu JW, Dong Q, Shi ZG, Hu HC, Wu DZ, Li LP. Effects of tending and thinning on non-structural carbon and stoichiometric characteristics of *Pinus yunnanensis*. J Beijing Univ. 2021;43:70–82.

[28] Wu JW, Liu S, Li JY. Photosynthetic and water consumption characteristics of afforestation tree species under drought stress in rocky desertification area of Guangdong Province. Acta Ecol Sin. 2016;36:3429–40.

[29] Wang XK, Huang JL. Principles and techniques of plant physiological and biochemical experiments. 3rd ed. Beijing, China: Higher Education Press. 2018.

[30] Bao SD. Soil agrochemical analysis. 3rd ed. Beijing, China: China Agricultural. 2000.

[31] McDowell NG. Mechanisms linking Drought, Hydraulics, Carbon Metabolism, and Vegetation Mortality. Plant Physiol. 2011;155:1051–9. 21239620 10.1104/pp.110.170704 PMC3046567

[32] Piper FI, Fajardo A. Foliar habit, tolerance to defoliation and their link to carbon and nitrogen storage. J Ecologx. 2014;102:1101–11.

[33] Yang B, Peng C, Harrison SP, Wei H, Wang H, Zhu Q, Wang M. Allocation mechanisms of non-structural carbohydrates of *Robinia pseudoacacia* L. seedlings in response to drought and waterlogging. Forests. 2018;9:754.

[34] Poorter L, Kitajima K. Carbohydrate storage and light requirements of tropical moist and dry forest tree species. Ecology. 2007;88:1000–11. 17536715 10.1890/06-0984

[35] Wei CY, Li YL, Jin ZX, Luo GG, Chen C, Shan FQ. Effects of shading on photosynthetic characteristics and non-structural carbohydrate content of *Heptacodium miconioides* seedlings. Bull Bot Res. 2022;42:1096–105.

[36] Sala A, Piper F, Hoch G. Physiological mechanisms of drought-induced tree mortality are far from being resolved. New Phytol. 2010;186:274–81. 20409184 10.1111/j.1469-8137.2009.03167.x

[37] Chen R, Han L, Zhao YH. Response of plant element traits to soil arsenic stress and its implications for vegetation restoration in a post-mining area. Ecol Indic. 2023;146:109931.

[38] Lu JY, Yang HM, Tian H, Zhang HS, Xiong JB, Liu Y. Effect of Water Addition on Carbon,Nitrogen and Phosphorus concentrations,and Stoichiometric Characteristics of Alfalfa Stems and leaves at different growth stage. Chn J Grassl. 2021;43:25–34.

[39] Wang K, Lei H, Wang ZY, Lv LY, Song LN. C, N and P distribution and stoichiometry characteristics of *Caragana microphylla* seedlings to drought stress. For Res. 2019;32:47–56.

[40] Liu JX, Zhang DQ, Zhou GY, Faivre-Vuillin B, Deng Q, Wang CL. CO_2_ enrichment increases nutrient leaching from model forest ecosystems in subtropical China. Biogeosciences. 2008;5:1783–95.

[41] Wang ZN, Yang HM. Response of ecological stoichiometry of carbon, nitrogen and phosphorus in plants to abiotic environmental factors. Pratacultural Sci. 2013;30:927–34.

[42] Yuan ZY, Chen HYH, Reich PB. Global-scale latitudinal patterns of plant fine-root nitrogen and phosphorus. Nat Commun. 2011;2:344. 21673665 10.1038/ncomms1346

[43] Zhang M, Ye L, Li SP, Cha XF, Dong Q. Effects of drought stress on the growth biomass and C,N,and P stoichiometric characteristics of *Fraxinus malacophylla* seedlings. J Sichuan Agri Uni. 2024;42:397–404.

[44] Bradshaw AD. Unravelling phenotypic plasticity why should we bother. New Phytol. 2006;170:644–8. 16684227 10.1111/j.1469-8137.2006.01761.x

[45] Nicotra AB, Atkin OK, Bonser SP. Plant phenotypic plasticity in a changing climate. Trends Plant Sci. 2010;15:684–92. 20970368 10.1016/j.tplants.2010.09.008

